# Global survey-based assessment of lifestyle changes during the COVID-19 pandemic

**DOI:** 10.1371/journal.pone.0255399

**Published:** 2021-08-13

**Authors:** Poonam Agarwal, Abhinav Kaushik, Sutapa Sarkar, Deepti Rao, Nilanjan Mukherjee, Vinita Bharat, Subhamoy Das, Amit Kumar Saha

**Affiliations:** 1 Department of Dermatology, Stanford University School of Medicine, Stanford, CA, United States of America; 2 Sean N. Parker Center for Allergy and Asthma Research at Stanford University, Stanford University School of Medicine, Stanford, CA, United States of America; 3 Gastroenterology and Hepatology, Stanford University School of Medicine, VA Palo Alto, Palo Alto, CA, United States of America; 4 Department of Microbiology and Immunology, Stanford University School of Medicine, Stanford, CA, United States of America; 5 Department of Neurosurgery, Stanford University School of Medicine, Stanford, CA, United States of America; 6 Department of Biochemistry, Stanford University School of Medicine, Palo Alto, CA United States of America; Columbia University, UNITED STATES

## Abstract

Along with the major impact on public health, the COVID-19 outbreak has caused unprecedented concerns ranging from sudden loss of employment to mental stress and anxiety. We implemented a survey-based data collection platform to characterize how the COVID-19 pandemic has affected the socio-economic, physical and mental health conditions of individuals. We focused on three broad areas, namely, changes in social interaction during home confinement, economic impact and their health status. We identified a substantial increase in virtual interaction among individuals, which might be a way to alleviate the sudden unprecedented mental health burden, exacerbated by general awareness about viral infections or other manifestations associated with them. The majority of participants (85%) lived with one or more companions and unemployment issues did not affect 91% of the total survey takers, which was one of the crucial consequences of the pandemic. Nevertheless, measures such as an increased frequency of technology-aided distant social interaction, focus on physical fitness and leisure activities were adopted as coping mechanisms during this period of home isolation. Collectively, these metrics provide a succinct and informative summary of the socio-economic and health impact of the COVID-19 pandemic on the individuals. Findings from our study reflect that continuous surveillance of the psychological consequences for outbreaks should become routine as part of preparedness efforts worldwide. Given the limitations of analyzing the large number of variables, we have made the raw data publicly available on the OMF ME/CFS Data Center server to facilitate further analyses (https://igenomed.stanford.edu/dataset/survey-study-on-lifestyle-changes-during-covid-19-pandemic).

## Introduction

The novel coronavirus, SARS-CoV-2 emerged in Wuhan, China, in early December of 2019 and is known to cause mild to severe respiratory illness when transmitted to humans [1]. It was previously considered to be droplet-borne and highly infectious due to several cases of human- human transmission through coughing, sneezing and nasal mucosa [2]. However, several recent studies have reported that SARS-CoV-2 virus particles are not only transmitted via droplets but also through air (airborne transmission) and cause infections [3–5].

The symptoms of the disease include high fever, cough, fatigue and shortness of breath [6], and is often accompanied by abdominal pain, diarrhea and nausea. Due to its similarity in the symptoms with Severe Acute Respiratory Syndrome (SARS), this novel infectious virus was named as SARS-CoV-2 and the disease was named as **Co**rona**vi**rus **D**isease 20**19** or COVID-19 [7].

COVID-19 outbreak started in the Hubei Province of Wuhan and took the shape of an epidemic in China by late January where numerous cases emerged at an alarming rate. There was a marked increase in new cases in USA, Europe and South Asian countries by late February, which prompted the World Health Organization (WHO) to declare it as a ‘Pandemic’. By mid-April 2020, at least 200 or more countries were affected with the virus, owing to its very high transmission rate. One of the major concerns of this disease is the asymptomatic transmission among individuals. Several infected individuals may show very mild to no symptoms for COVID-19 disease, but are still capable of spreading it to other individuals [8, 9]. Preliminary studies and several data reports suggest that this virus infects people with comorbidities (one or more chronic diseases) and old people more aggressively, than people of younger age [10]. A study in Nature Medicine showed that the number of cases in children was low as compared to the adults from the data collected from China, Italy, Japan, Singapore, Canada and South Korea [11]. Even though COVID-19 hospitalizations and death can occur in young and middle-aged adults, people of age 60 years or older are subject to higher risk [12]. Also, several reports suggest that adults and old people with pre-existing medical conditions like diabetes, kidney disease, cardiovascular disease, chronic obstructive pulmonary disease, hypertension and several other chronic illnesses are more severely affected [13–16]. With the progression of this pandemic since over an year, emerging studies suggest that the novel variants of SARS-CoV2 virus including the B.1.1.7 variant may be significantly more transmissible and infectious outcompeting the preexisting variants [17].

The consequences of the COVID-19 pandemic has not only incurred a worldwide negative impact on the socio-economic status but also threatened people’s lives and caused high mortality rates in an unanticipated manner [18]. The prevalence of infection, patient surges and death rates eventually led to situations of lockdown or shelter-in-place practices in different countries, thereby introducing an unforeseen challenge in the daily lives of people across the globe [19, 20]. As a safety measure to prevent the spread of COVID-19, social distancing and home isolation strategies including closing schools, offices, factories and other public places had been adopted globally. These strategies have proven to reduce cross-infection effectively [21]. However, as humans have evolved to be socially connected, these long periods of confinement have influenced an individual’s life in different ways based on one’s situations and the dwelling environment. For instance, the effect of lockdown/shelter-in-place has caused not only mental health burden in common people worldwide, but also has affected some of them financially. Due to the lockdown policies, the government of respective countries had to close schools and colleges, lay off individuals from jobs or overwork healthcare workers for treatment of the infection surge. These unprecedented actions have led to an inevitable change in social practices and norms. In this era of globalization, an abrupt change in the fast-paced lifestyle to a sedentary lifestyle has created both positive and negative effects on the common population. While a few people have discovered new hobbies, habits, several others have been a victim of anxiety and depression, especially for young adults. While there have been substantial investigation into understanding the physiological aspects to develop diagnostics [22] and vaccines [23], the socio-psychological impacts of the pandemic remain understudied. Here we present the findings from our study to investigate how the global lifestyle changes have affected the socio-economic, physical and mental well-being during the ongoing COVID-19 pandemic. Given the limitations of analyzing the large number of parameters, we have made the raw data publicly available on the OMF ME/CFS Data Center server. (*https*:*//igenomed*.*stanford*.*edu/dataset/survey-study-on-lifestyle-changes-during-covid-19-pandemic*).

## Materials and methods

Sample and Study Design: This study was based on a series of survey-based questions conducted in the period between June 10 and August 5, 2020. A 3-months online questionnaire survey was conducted on the REDCap software, a secure and widely used platform for creating custom modules to post online questionnaires and collect data worldwide. The online survey link was circulated through a standard study invitation message within known acquaintances, social media and communication platforms such as email, Linkedin, Facebook, Instagram, Twitter and WhatsApp. Only individuals above the age of 18 years were allowed to participate in the study. There were no other exclusion criteria.

We applied the principle of maximum diversity to recruit a representative sample for this study. The survey was eligible for participants who are 18 years old or above. As an effort to sustain maximum representativeness and in order to keep the survey unbiased, the participants could belong to any geographical location. A total of 3253 responses were collected across 47 countries using the RedCap survey link (Table 1). After excluding responses that met the exclusion criteria (age <18 years), duplicates and invalid entries, the final data included 2683 participants.

**Table 1:** List of countries where the survey participants were located, with the corresponding number of participants per country.

Country	Total entries	Completed entries	Country	Total entries	Completed entries
United State of America	2283	1991	Netherlands	5	3
India	372	266	Spain	4	3
New Zealand	93	79	Slovakia	3	3
Australia	78	65	China	5	2
Canada	61	40	Saudi Arabia	3	2
Italy	52	34	Israel	2	2
France	31	25	Singapore	2	2
Nepal	37	21	United Arab Emirates	2	2
United Kingdom	22	19	South Africa	3	1
Indonesia	22	16	Ukraine	3	1
Pakistan	21	14	Papua New Guinea	2	1
Hungary	15	12	Poland	2	1
Germany	12	11	Slovenia	2	1
Croatia	14	10	Switzerland	2	1
Mexico	10	10	Turkey	2	1
Malaysia	8	7	Algeria	1	1
Brazil	8	6	Austria	1	1
Bosnia and Herzegovina	7	6	Bangladesh	1	1
Sri Lanka	6	4	Egypt	1	1
Luxembourg	5	4	Liberia	1	1
Greece	4	4	Monaco	1	1
Taiwan	7	3	Romania	1	1
Uganda	1	1	Sweden	1	1
Afghanistan	17	0	Tanzania	1	1
Angola	3	0	Burundi	1	0
El Salvador	2	0	Christmas Island	1	0
Akrotiri	1	0	Ireland	1	0
American Samoa	1	0	Japan	1	0
Antarctica	1	0	Portugal	1	0
Belgium	1	0	South Korea	1	0
Burundi	1	0	Zimbabwe	1	0

The questionnaire included three domains/sections: demographic information (such as age, sex, living areas, education and occupation), hobbies and habits, effect on socio-economic lives and mental health/disease awareness. All analyses were performed using R software with gg plot package. A majority of the final plots were made using GraphPad Prism. This was primarily an observational study, and hence detailed statistical analyses were outside the scope of this manuscript.

The study was approved by the Stanford University Institutional Review Board (Protocol 56465; Exempt). Only participants over the age of 18 were considered for the study. Informed consent was obtained for study participation from all the participants. Written consent was obtained electronically prior to the start of the survey. The survey can be found here: https://is.gd/COVIDSocialSurvey.

All the raw data are available on the OMF ME/CFS Data Center server, including data collected after August 5, 2020 (https://igenomed.stanford.edu/dataset/survey-study-on-lifestyle-changes-during-covid-19-pandemic). Readers can perform subsequent analyses on the available data, to shed further light on the different parameters. The survey link is still active, but there has been no active promotion of the study beyond the mentioned date range.

## Results and discussion

As of August 5, 2020, there were 2683 valid, questionnaire entries which comprised 82.4% of the total participants who initiated the survey (Table 1). The online survey method of nonprobability sampling was used to recruit participants via social media posts that targeted the general adult population (aged >18 years old).

**Fig 1:**
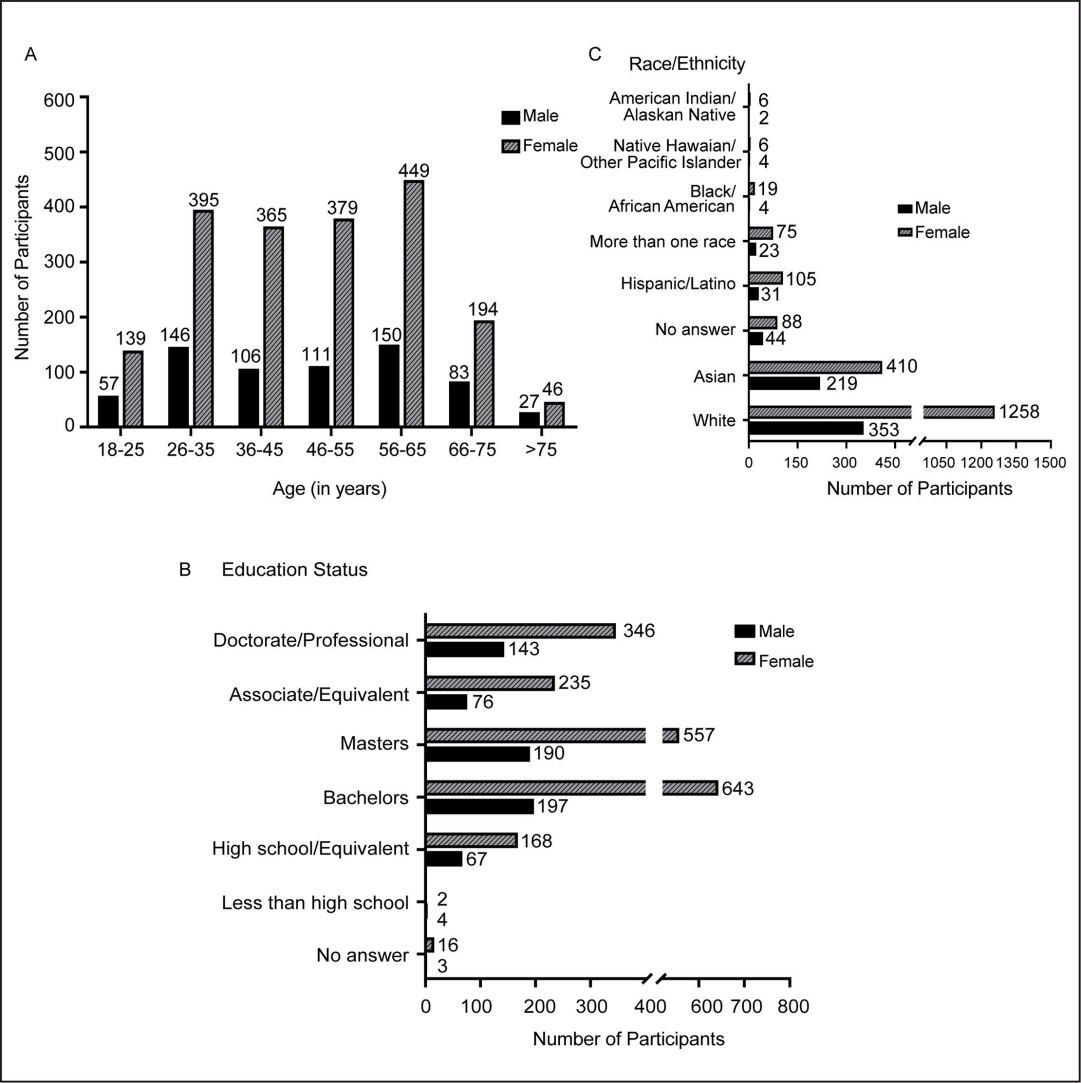
Graphical representation of demographics of the study participants. Age-distribution of the participants. B. Educational status of the participants. C. Racial/ethnic background of the participants. All the plots are further subdivided by gender.

This sample population was composed by anonymous denizens of 47 countries, with the highest number of participants from the United States, India, New Zealand and Australia (Table 1). Age-wise profiling of the survey takers show that 22.7% of the participants belonged to the age group between 56–65 years alone, followed by 26–35 years (20.6%) age group. Individuals under the age groups of 36–45 years and 46–55 years also formed a significant portion (36%) of the survey participants (Fig 1A). The rest were either adults younger than 25 years old (7.6%) or senior citizens above 65 years old (13.1%). The majority of participants were female (1967/2683) and approximately one- fourth of the total participants were male (680 participants). There were also a small number of participants who identified as transgenders (6 people), other (8), and 22 participants chose not to disclose their gender. Since the number of participants who identified as male or female was much larger, our analyses only consider these two categories. The educational status for most of these participants ranged from an undergraduate to graduate degree as their highest qualification with 88.9% of the people having a bachelor’s degree or higher (Fig 1B). The survey takers had the following racial/ethnic identities: White, Asian, Hispanic or Latino, Black or African American and “others” such as Native Americans, Hawaiians, Pacific Highlanders, Alaskan Native, or some with more than one racial identity. The majority of the participants were either White (60.4%) or Asian (23.6%), whereas 16% participants fell under the above mentioned “other” categories (Fig 1C). For each of these categories, distribution for male vs. female participants is represented in the plots.

**Fig 2:**
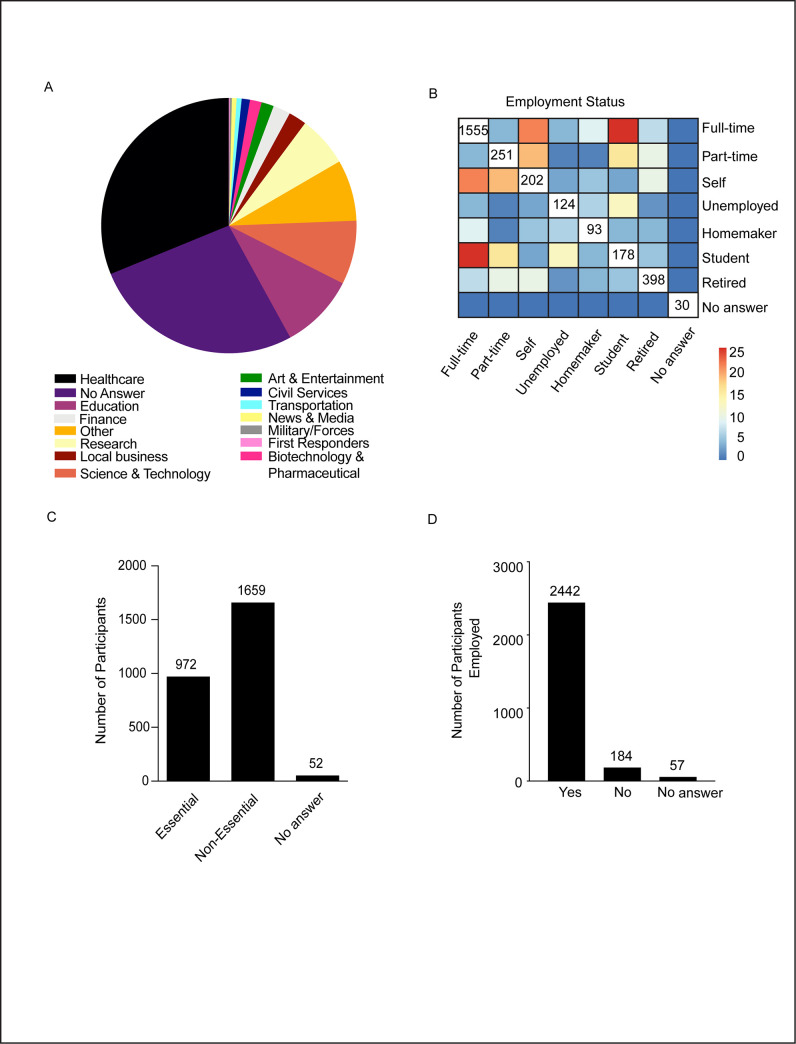
Professional background of the participants. A. Classification of employment categories. B. Employment status of the participants during the study period. C. Distribution of individuals on the basis of essential or non-essential workers. D. Participants’ employment retention status during the study period.

To gain insights on the impact of the COVID-19 pandemic on professional lives, we asked the occupational background of the survey takers by listing a range of employment categories or occupational groups including art and entertainment, finance, research, first responders, healthcare, news and media, transportation, civil and military services and other relevant staff. The highest percentage of our survey takers belonged to the healthcare sector (31.2%). About 26% of the survey takers opted not to disclose their occupational category. The next three highest representations were from education (9.5%), science and technology (8.1%) and research sectors (6.4%) (Fig 2A). Furthermore, to analyze how their occupational lives were influenced by the unprecedented changes in the routine, we asked what best reflected the survey takers current professional status. Fig 2B is a heat map looking into the current employment status, taking into account potential overlaps between different professional statuses. The colored grids represent overlaps between any two given categories. For the non-overlapping population, represented along the diagonal, we have specified the exact number of participants in the figure. For the participants who have two professions, we represented the numbers using a color scale. The numerical values in the diagonal grids represent the total number of participants in individual categories. Even though there was a rapid shift to remote working options, most of the participants were employed full-time followed by a second highest group of retired personnel who took our survey. A smaller subset of the participants belonged to part-time or self-employment status followed by students, unemployed members and homemakers (Fig 2B). Thus, compared to other occupational groups, more than 50% of the survey takers belonged to full-time research and healthcare segments. Moreover, we found some overlap between the different categories. For instance, most of the full-time workers were students or self-employed individuals (Fig 2B). A critical separation of the workforce during the time of a pandemic generated crisis is classifying the essential and non-essential workers. Responses for this survey question revealed that there were 1659 essential workers present in our study cohort (61.8%) and 972 workers (36.2%) belonged to the non-essential workers category, with a few participants (n = 52, 1.9%) who opted not to answer this question (Fig 2C). To understand whether this study group experienced unique challenges such as risk of financial stability during the COVID-19 crisis through loss of employment we asked the participants their current occupational status. Most of the candidates (91%) who took the survey were not affected by loss of employment except for a small subgroup (6.85%) who lost their jobs (Fig 2D). Thus, data from this section of the study suggests that there was a mixed distribution of candidates with regard to their occupational status and a major fraction of them did not lose employment.

**Fig 3:**
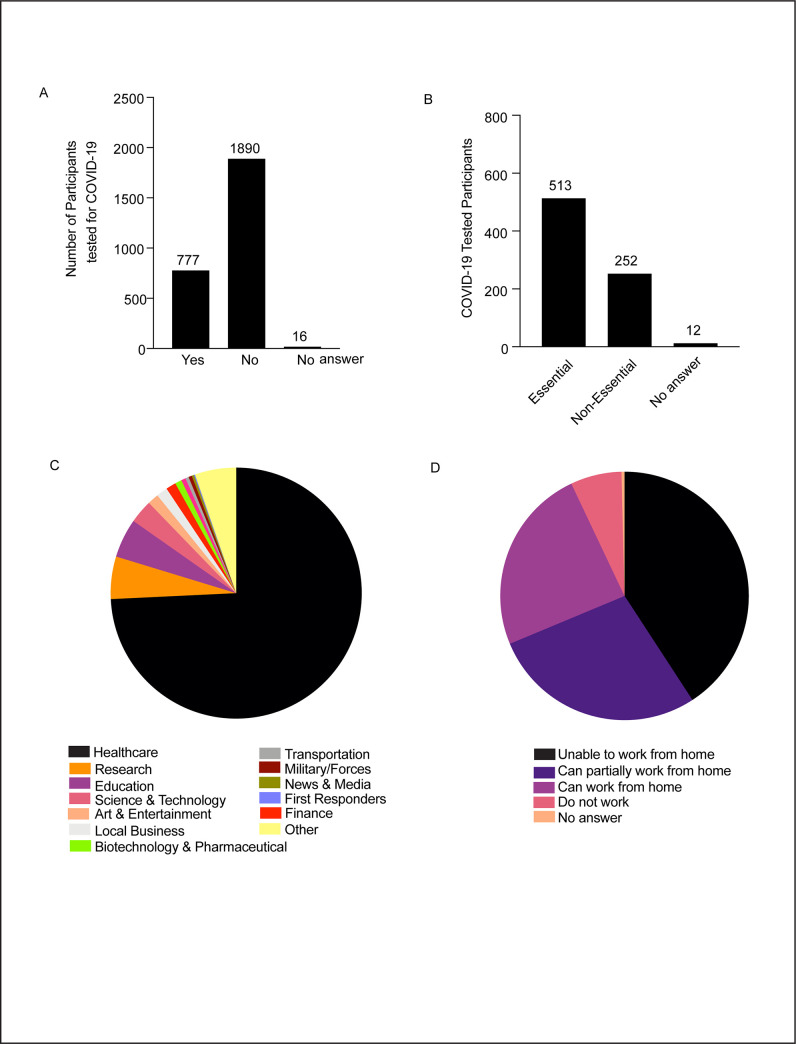
COVID-19 testing status and professional background of the test takers. A. Distribution of participants based on COVID-19 testing. B. Classification of COVID-19 test takers into essential and non-essential workers. C. Occupational categories of COVID-19 test takers. D. Classification of tested individuals on the nature of work based on physical location.

We then assessed whether the study participants were tested for COVID-19 and found that a little more than one-fourth of the total participants, i.e., n = 777 or 29% took the COVID-19 detection test and the majority 1890 or 70.4% of total participants did not test for an infection until the time of filling the survey (Fig 3A). In order to estimate whether it was mandatory for this subset of COVID-19 test takers to be tested, we analyzed if they were essential or non-essential workers. The results showed that a large number of the tested individuals were essential workers (511 out of 777 i.e., 65.7%) and a few of them (244 out of 777 i.e., 31.4%) belonged to the non-essential workers category while 1.5% preferred not to answer (Fig 3B). This result suggests that along with the essential workers for whom COVID-19 testing was expected to be mandatory as a safety precaution, there was an alarming health concern among the general public /non-essential workers to obtain the COVID-19 test and thereby assure that they have not been exposed to an infection. Furthermore, a comparative analysis also informed what fraction of the total essential and non-essential workers in our study group took the COVID-19 test. A little more than half of the total essential workers, 511/972 i.e., 52.5% took the test while working as frontline taskforce which accounts for 511/2683 or 19% of the total survey participants. As expected, this fraction was lower in the non-essential workers group where only 252/1659 i.e., 15.1% were tested (S1 Fig). We then assessed whether the sub-population who opted for taking the COVID-19 detection test belonged to a few of the specific occupations or were evenly distributed among all categories. Upon analyzing which occupational categories these COVID-19 test takers belonged to, we found that the majority of them (67.3%) were healthcare workers followed by employees from the research and education sector (9.5%) (Fig 3C). To measure how people from different employment backgrounds were impacted by the unexpected and abrupt restrictions such as lower density of occupants in the office and business spaces we asked whether their jobs could be performed remotely. A vast majority of the tested individuals did not have the flexibility of working from home since most of our study cohort were employed by healthcare or research/education organizations. About 40.1% of the participants had to be onsite at their workplaces and 28% people reported that their work could only be partially done from home. Only 24.2% of the total participants could work entirely remotely with having any adverse effect on their work (Fig 3D). Overall, these studies show that regular monitoring through periodic testing for workers in healthcare and related sectors could help circumvent the spread of COVID-19 disease allowing smooth functioning of the healthcare delivery systems when working remotely is not an option.

**Fig 4:**
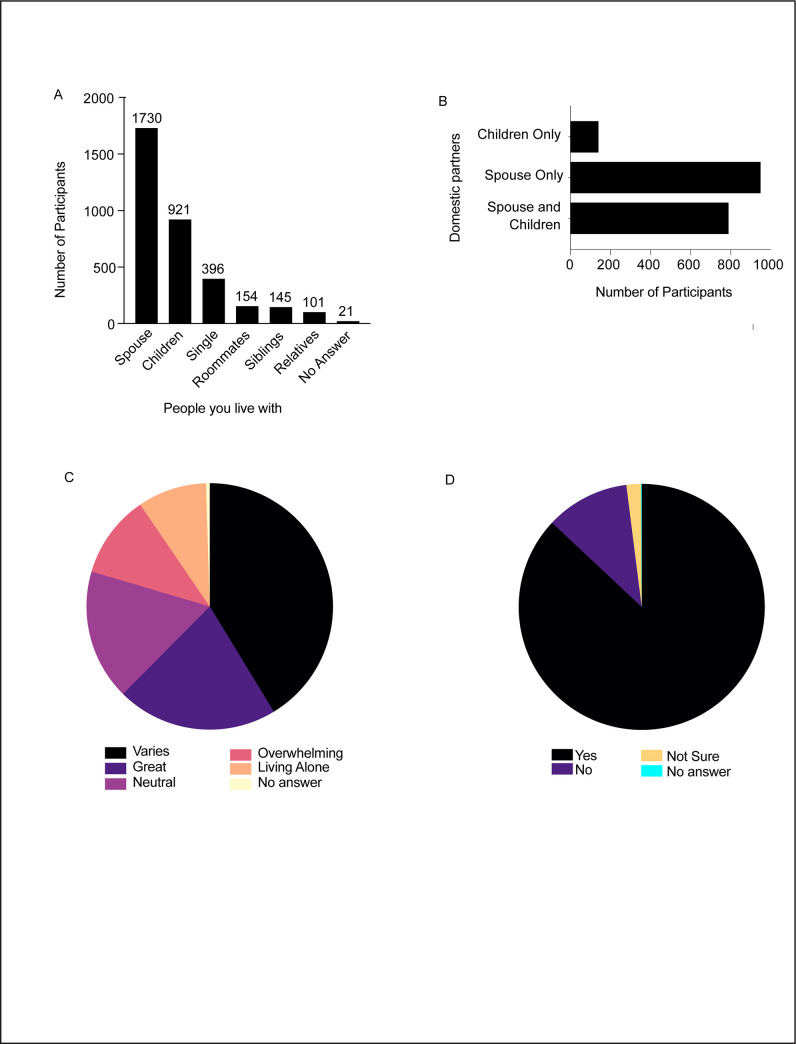
Impact of home isolation strategies implemented during the study period. A. Cohabiting profile of the study participants. B. Overlap between individuals who lived together with spouse and/or children. C. Effect of spending more time in the household as opposed to usual lifestyle on the participants’ mindset. D. Changes in social interaction of the study participants during the study period.

Previous studies have shown that even under normal circumstances our overall well-being is influenced by the people we surround ourselves with [24]. During the COVID-19 pandemic lockdown when everyone experienced either shorter or longer periods of home isolation, the people we live with is an important determinant of our social and mental wellbeing. Therefore, we designed our survey questionnaire to assess the immediate household cohabitants of the study participants and to explore how it impacted their daily routines. Our results show that the majority, ~65% (1730/2683) of the survey participants lived with their spouses while others had roommates, siblings or relatives as companions, and a few lived by themselves. Single individuals formed the second highest category (14.7%). There was almost an equal distribution of individuals living either with their sibling(s), other relatives or roommates (~6%) while 1% of the entries preferred not to answer this question (Fig 4A). Based on further analysis of participants who lived with their spouse, we found that 45% among them (781/1730) had children in their household (Fig 4B). A comparison of the overlapping categories revealed that most of our study participants lived with one or more companions during this challenging time and only a small percentage lived by themselves (14%). In terms of how people felt about spending more time indoors with other household members during the pandemic, there were mixed reactions ranging from fluctuation in their opinion to feeling great or neutral (Fig 4C). The COVID-19 pandemic altered social interactions among people around the world. In particular, more than 2/3^rd^ of the participants experienced a significant change in the extent of social interaction with people not living in their common household (Fig 4D). There was a pronounced increase (87.1%) in the level of social interaction during the isolation phase and only a few individuals (10.8%) responded to have no change in their interactions with other people.

**Fig 5:**
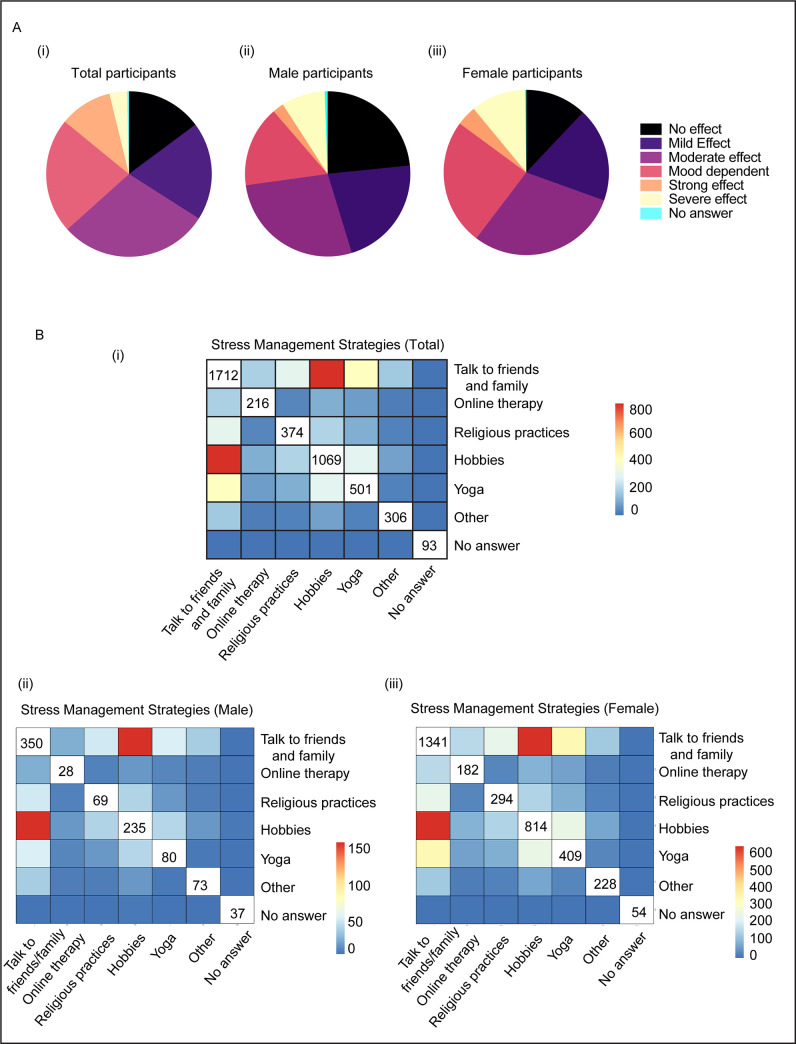
Effect on mental health of the study participants. A. Extent of mental health effect due to home isolation during the study period. (i: Total number of participants; ii: Male participants; iii: Female participants). B. Stress management strategies adopted by individuals. (i: Total number of participants; ii: Male participants; iii: Female participants).

The unprecedented COVID-19 disease outbreak, which led to serious health concerns, uncertainty and havoc in the global community, could be overwhelming and cause strong emotions in people of all ages [25–27]. Although preventive measures adopted through public health actions such as social distancing are necessary to reduce the spread of the disease, these are associated with fear, loneliness and anxiety in the people. We wondered how people were adjusting to the changing environment with the sudden reduction in out-of-home activity and how it affected their mental health. Therefore, in our global survey, we chose to ask about people’s outlook towards the coronavirus crisis by creating different categories to gauge the extent of mental health changes ranging from none to moderately or extremely affected. There were mixed responses and opinions for how the participants felt under these changing circumstances. While around 30% of the participants stated that they were moderately affected, 13.5% people faced extreme and overwhelming mental health challenges (Fig 5A(i)). Moreover, several recent studies suggest that the current pandemic has impacted the physical and mental health of men and women differently [28]. This is consistent with our analysis of the psychological and behavioral reactions to the COVID-19 pandemic in the male and female subgroups, where females were found to be more affected than their male counterparts and a larger proportion considered these effects to be mood dependent (Fig 5A (ii, iii)).

We also found that both the essential and non-essential workers groups had similar effects of social isolation on their mental health. Our current findings and similar recent studies [29, 30] have shown that the unprecedented disease outbreak and the lockdown phase has induced higher chronic stress and psychological distress in the human community at large. We therefore aimed to assess the stress management strategies adopted by our study participants and how well they coped with their mental health burden. We focused on a few options that are considered as the prime coping strategies used by individuals facing stressful situations [31]. These include pursuing hobbies, physical activity such as yoga, online therapies, religious practices and speaking to friends and family. As a way to cope with the accumulating stress and anxiety, there was a marked increase in the frequency of interactions with friends and family members (63.8%) as well as exercising or pursuing individual hobbies (58.5%). Moreover, we observed a correlation that the individuals who pursued hobbies were also engaged in social interactions more than others. There were a very few people, at least in this study cohort, who used online therapies (8%) or religious practices (13.9%) (Fig 5B(i)). While being required to isolate themselves at home and prohibit physical social gatherings, we found that individuals actively discovered alternate socializing ways through virtual interactions. We then categorized the male and female participants separately and found slight differences in the response strategies between these subgroups. For instance, activities like practicing yoga and pursuing hobbies were more prevalent in the females as compared to the male participants (Fig 5B (ii, iii)). Thus, data from this section of the study suggest that an individual’s level of virtual interaction with family, friends and acquaintances increased while going through this phase of severe infectious disease prevalence. Overall, the majority of the survey takers were employed and living with one or more companions and yet chose social interaction, in part, as a coping mechanism to relieve their stress and anxiety during the COVID-19 pandemic.

**Fig 6:**
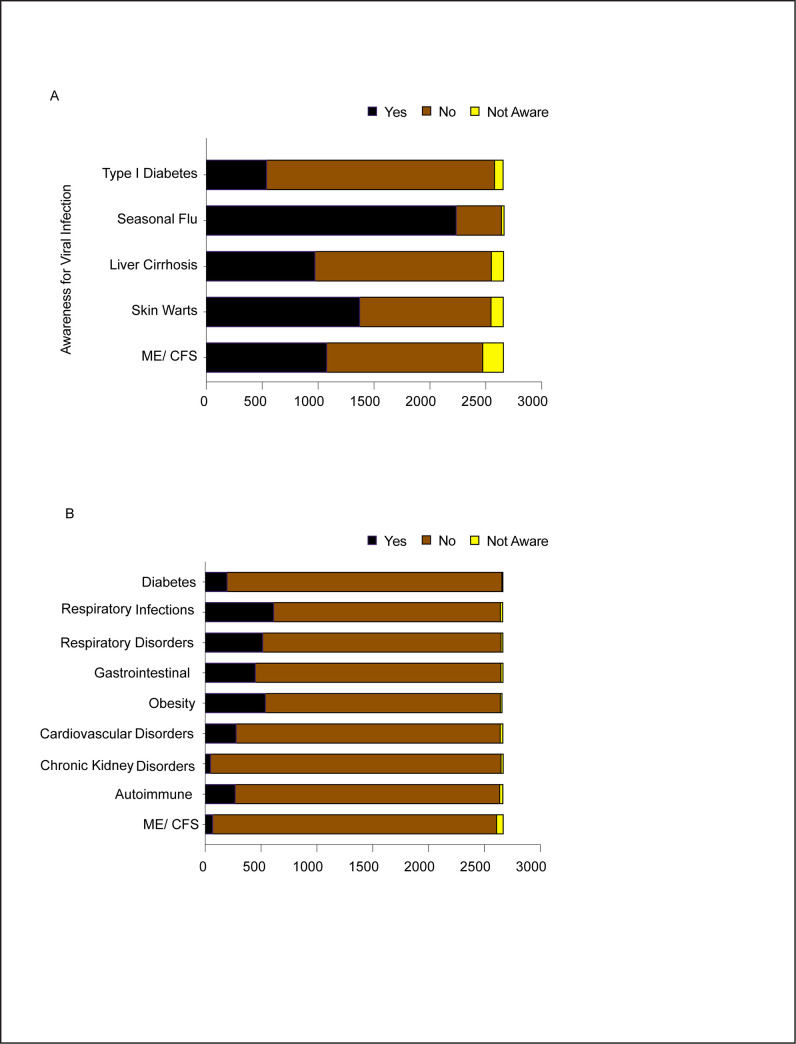
Public health aspects of the study. A. Awareness of viral infection associated diseases among the study participants. B. Number of survey takers who had underlying conditions before the COVID-19 outbreak.

Since the COVID-19 pandemic presents a major threat to public health, it is necessary for the human population to acquaint ourselves with some awareness of viral infections and the diseases associated with it. Viral infections can cause a series of disorders affecting multiple organs in the body including the lungs, liver, gut, brain, heart, pancreas and kidneys [32]. In order to estimate, as well as raise general awareness about viral infections or other manifestations associated with it we asked our study participants from different demographic and occupational backgrounds about their idea on the disorders that a viral infection might cause. The majority of the survey takers were aware of the impact of viral infection of seasonal flu. This information is particularly useful as we approach the annual flu season. Among our survey takers, the awareness was least that viral infections can cause Type 1 Diabetes. About 40–60% survey takers were familiar that viral infections can cause skin warts, Liver Cirrhosis, and Myalgic Encephalomyelitis/Chronic Fatigue Syndrome (ME/CFS; an unexplained, chronic disease that affects millions worldwide [33, 34]) (Fig 6A). Of note, there is an emerging body of evidence indicating that a section of the COVID-19 patients, especially the long haulers, will eventually develop ME/CFS [35, 36]. To find whether the survey takers had other underlying health conditions that might exacerbate the effects of COVID-19, we shortlisted a few disorders that are believed to have that effect. About 6.6%, 9.54%, 17.35%, 19.53%, 16.42%, 14.47%, 9.39%, 2.45% and 4.19% of our respondents suffered from diabetes, cardiovascular disorders, obesity, respiratory infections, other respiratory disorders, gastrointestinal disorders, autoimmune diseases, Chronic kidney disorders, and ME/CFS, respectively. Overall, the majority of our respondents did not have the stated underlying conditions that could exacerbate their COVID-19 risk (Fig 6B).

### Limitations

Although our survey design encompasses various dimensions to estimate the impact of the COVID-19 outbreak on the participants, we cannot provide an exhaustive analysis of all the possible variables here, as that is beyond the scope of a single manuscript. To overcome these shortcomings, we have made all the raw data available for the community to conduct further analyses (https://igenomed.stanford.edu/dataset/survey-study-on-lifestyle-changes-during-covid-19-pandemic). As an example of such analysis, we have provided a second-degree analysis of some of the parameters for the survey-takers who experienced changes in their social interaction (S3 Fig).

## Conclusions

Our web-based study indicates that there was a moderate to severe effect on an individual’s social, financial and mental health conditions during the COVID-19 disease outbreak. Our data does not show that there is significant variance in peoples’ outlook and behaviors across countries. Among the survey completers (n = 2683), 22.7% of the participants belonged to the age group 56–65 years while 56.6% were 26–55 years old and 78.24% held a bachelor’s degree or above. More than 50% of these survey takers belonged to full-time research and healthcare segments. We identified that most of our study group members did not lose their jobs and one reason that we do not observe significant loss of employment in our study could be the fact that a greater fraction of the survey takers was from the healthcare sector and research area, and a large percentage among them identified themselves as essential workers. The essential workers comprised the most significant portion of the small population of COVID-19 test takers in our entire study. Since they were healthcare workers and essential, they were not remote workers. Interestingly, despite retaining their jobs, the majority of our survey takers indicated change in their social interaction. Participants were engaged in an increased frequency of technology-aided distant social interaction, focus on physical fitness and leisure activities were adopted to serve as a coping mechanism during this period of home isolation. Moreover, a large portion of the study participants had a general awareness about viral infections or other manifestations associated with it. Collectively, these metrics provide a succinct and informative summary of the socio-economic and health impact of the COVID-19 pandemic on the individuals. In conclusion, our findings provide data support for understanding people’s state of mind during an unexpected period of social isolation associated with such a pandemic. Findings from our study reflects the fact that continuous surveillance of the psychological consequences for outbreaks should become routine as part of preparedness efforts worldwide.

## Supporting information

S1 Fig Group wise distribution showing the fraction of total essential and non-essential workers who took the COVID-19 test. (TIF)

S2 Fig Comparative analyses of essential vs non-essential workers. A. Comparison with regard to their employment status during the study period. B. Change in social interaction levels. As anticipated and shown in S2 Fig. A, we found that the non-essential group lost more jobs as opposed to the essential workers. Moreover. there was a bigger effect on social interaction among the non-essential working group as compared to the essential workers (S2B Fig).
(TIF)

S3 Fig Comparative analyses between the two groups of participants who experienced changes in their social interaction and those who reported no changes in social interaction during the study period. The comparisons were based on their: A. Employment status, household companion and COVID-19 test status. B. Mental health status of participants. C. Strategies to cope with mental health issues. For all of the above metrics, there was similar distribution in the two groups as depicted by the bar graphs (S3A Fig). We then evaluated what effect these two groups of survey takers had on their mental health as a result of COVID-19 and social isolation. In particular, we asked about the extent of impact on their mental health by providing different levels or categories to choose from, such as, extremely, moderately to overwhelming or no effect at all. Our analysis shows that a large portion of the social interaction affected individuals had moderate effects on their mental health whereas the majority of the participants who did not have an effect on their social interaction neither had any kind of mental health impact (S3B Fig). Moreover, a substantial proportion of the participants who had changes in their social interaction felt that the overall quality of their mental health could have been better whereas the other group had a neutral opinion. We also measured the differences with regard to stress coping mechanisms in these two groups of individuals. Our data reflects that both these groups had a similar trend of involvement in alternate activities as a way to cope with stress and anxiety. For instance, in both the groups, the largest fraction of people opted to communicate with their friends and family the most followed by pursuing their hobbies (S3C Fig).
(TIF)

S1 File Survey questionnaire used for this study. Also available online at https://is.gd/COVIDSocialSurvey.
(DOCX)
