# Supplementary figures and images for: Global survey-based assessment of lifestyle changes during the COVID-19 pandemic
Source: PLoS One. 2021 Aug 13;16(8):e0255399. doi: 10.1371/journal.pone.0255399 (PMC8362972; doi:10.1371/journal.pone.0255399)

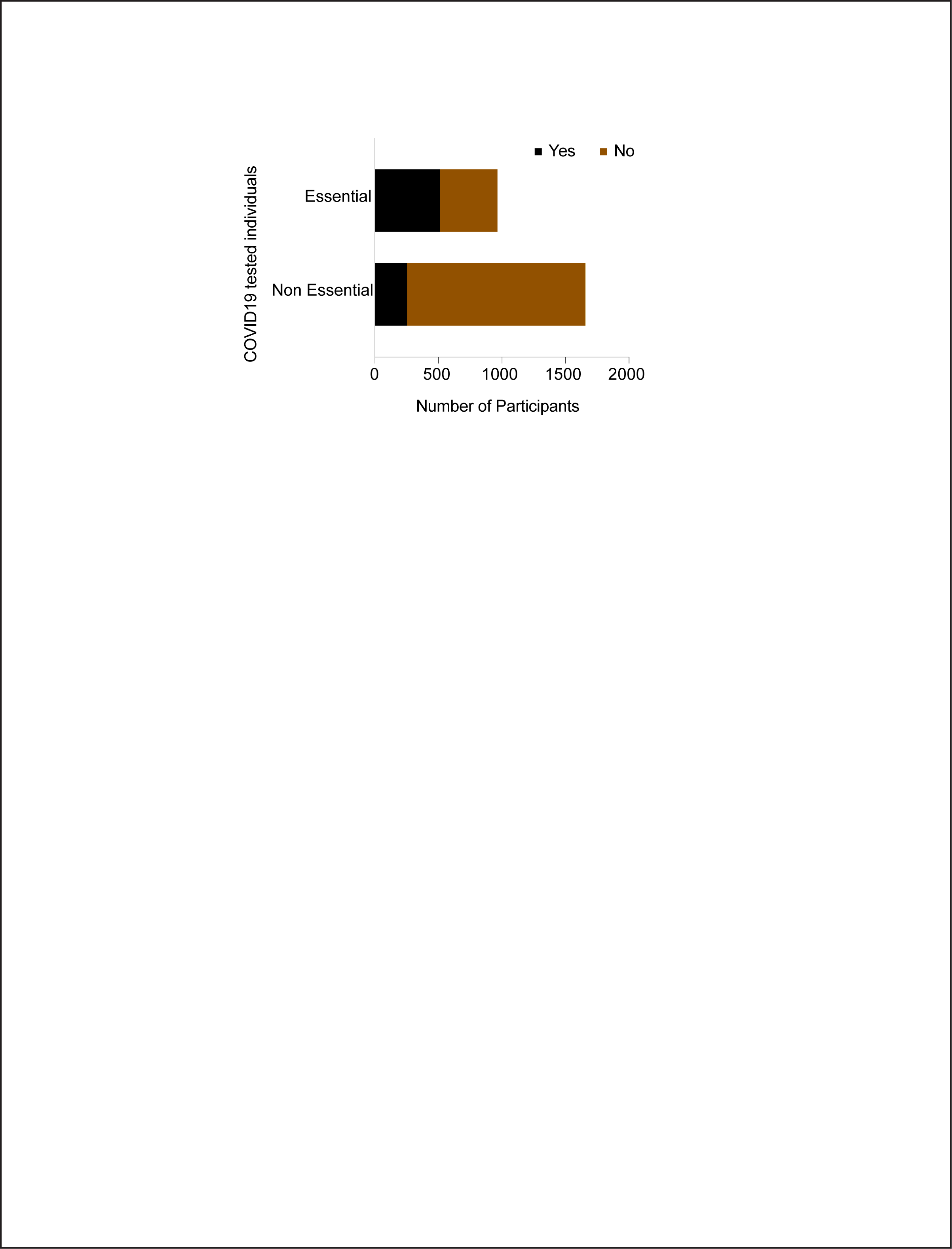

Supplement: S1 Fig — (TIF) [file pone.0255399.s001.tif]

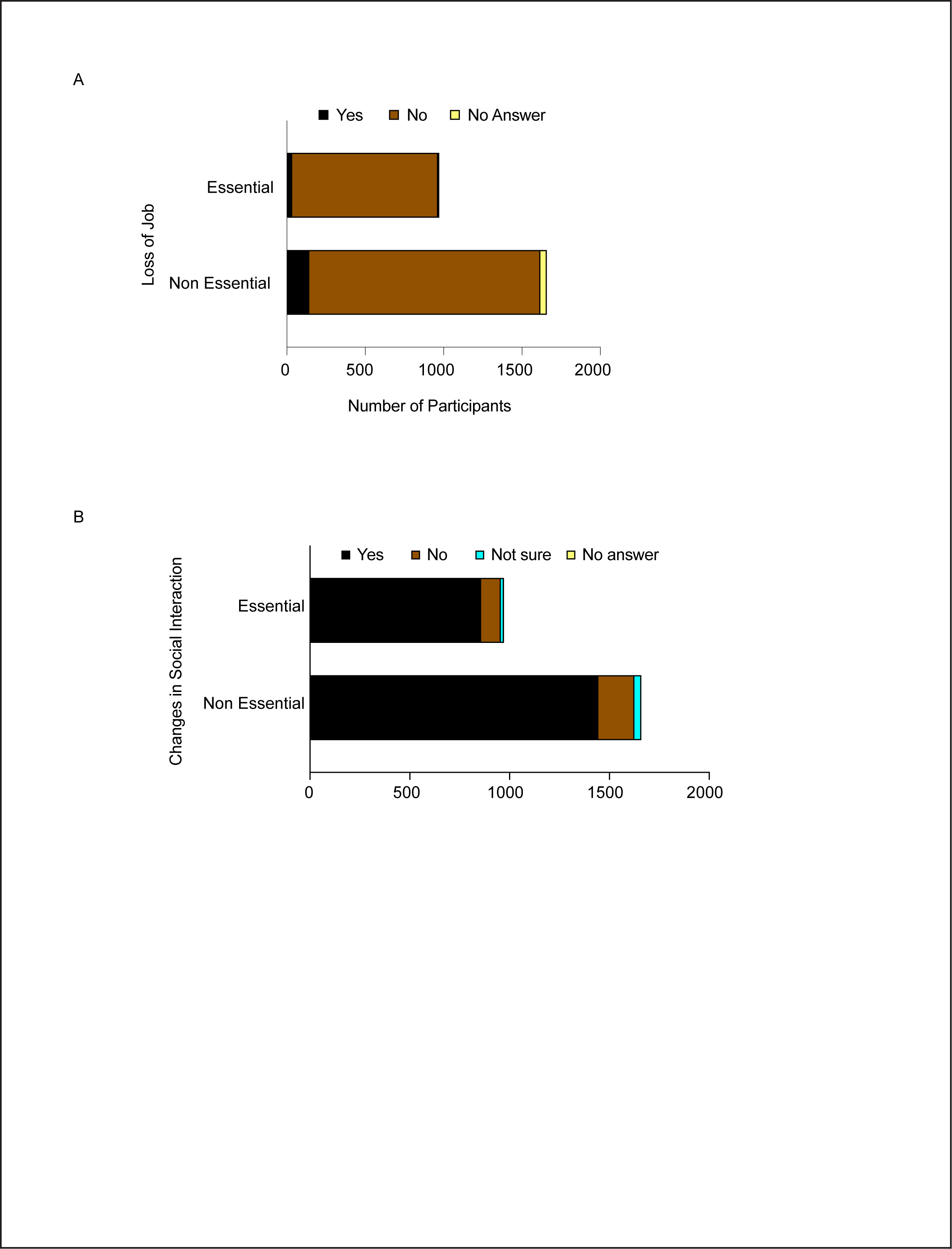

Supplement: S2 Fig — A. Comparison with regard to their employment status during the study period. B. Change in social interaction levels. As anticipated and shown in S2 Fig. A, we found that the non-essential group lost more jobs as opposed to the essential workers. Moreover. there was a bigger effect on social interaction among the non-essential working group as compared to the essential workers (S2B Fig). (TIF) [file pone.0255399.s002.tif]

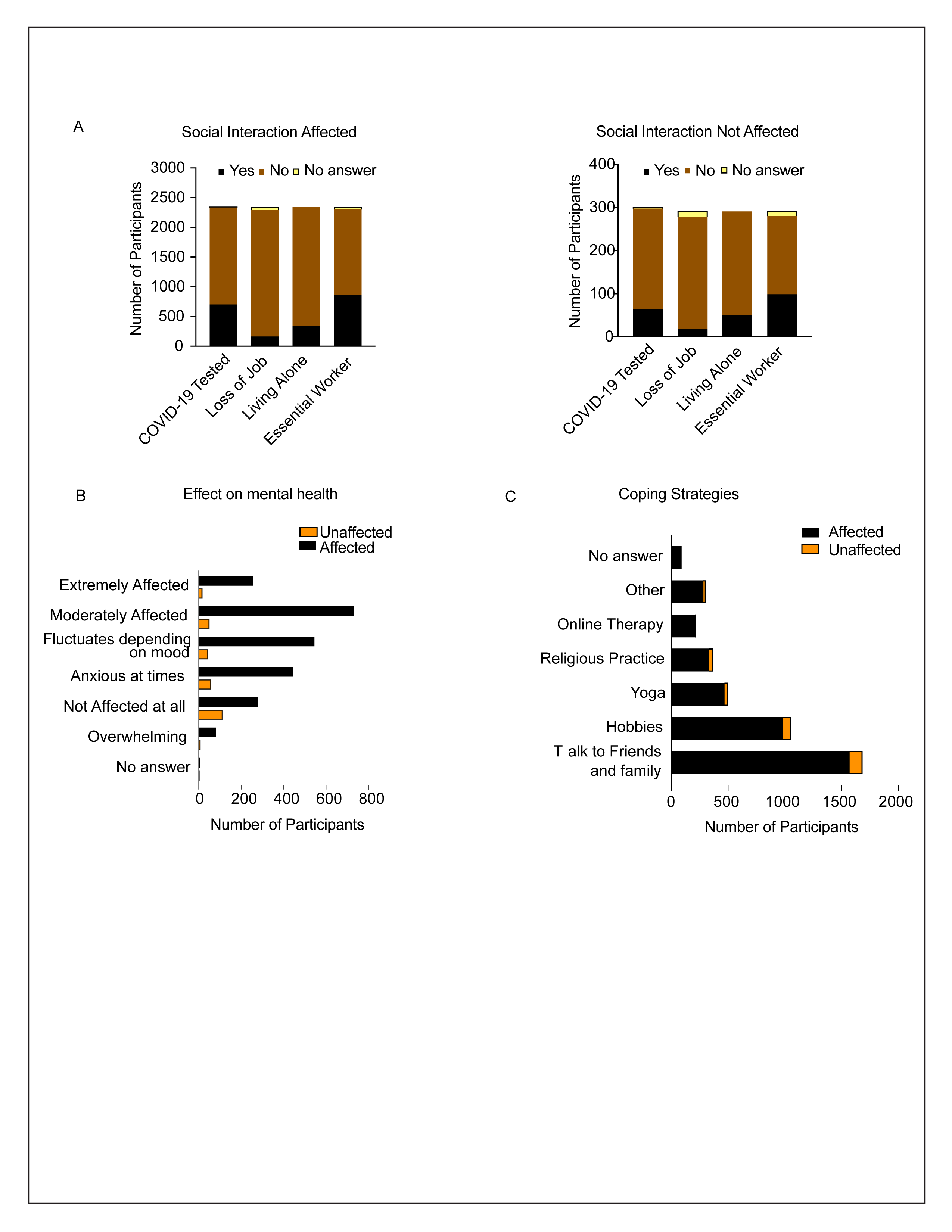

Supplement: S3 Fig — The comparisons were based on their: A. Employment status, household companion and COVID-19 test status. B. Mental health status of participants. C. Strategies to cope with mental health issues. For all of the above metrics, there was similar distribution in the two groups as depicted by the bar graphs (S3A Fig). We then evaluated what effect these two groups of survey takers had on their mental health as a result of COVID-19 and social isolation. In particular, we asked about the extent of impact on their mental health by providing different levels or categories to choose from, such as, extremely, moderately to overwhelming or no effect at all. Our analysis shows that a large portion of the social interaction affected individuals had moderate effects on their mental health whereas the majority of the participants who did not have an effect on their social interaction neither had any kind of mental health impact (S3B Fig). Moreover, a substantial proportion of the participants who had changes in their social interaction felt that the overall quality of their mental health could have been better whereas the other group had a neutral opinion. We also measured the differences with regard to stress coping mechanisms in these two groups of individuals. Our data reflects that both these groups had a similar trend of involvement in alternate activities as a way to cope with stress and anxiety. For instance, in both the groups, the largest fraction of people opted to communicate with their friends and family the most followed by pursuing their hobbies (S3C Fig). (TIF) [file pone.0255399.s003.tif]
