# Supplementary material for: Global survey-based assessment of lifestyle changes during the COVID-19 pandemic
Source: PLoS One. 2021 Aug 13;16(8):e0255399. doi: 10.1371/journal.pone.0255399 (PMC8362972; doi:10.1371/journal.pone.0255399)
Supplement: S1 File — Also available online at https://is.gd/COVIDSocialSurvey. (DOCX) [file pone.0255399.s004.docx]

**The survey questions are as follows:**

# Global Survey of Lifestyle Changes during COVID-19 Pandemic

There are 5 categories in this survey:

Introduction, Social interactions, Hobbies & Health, Professional life, Social contributions. This survey is only for adults >18 years of age.

If you are not able to finish survey now, you can save and come back later.

Once you click submit at the bottom of the page, it will automatically take you to next section. Part 1 out of 5

**Consent to Participate**

DESCRIPTION: You are invited to participate in a research study on effect of lifestyle changes due to COVID-19 pandemic. You will be asked to answer a series of questions below.

TIME INVOLVEMENT: Your participation will take approximately 10 minutes.

RISKS AND BENEFITS: The risks associated with this study are none. The benefits which may reasonably be expected to result from this study are understanding of lifestyle changes, impact on mental and physical well-being, effect on social contributions. We cannot and do not guarantee or promise that you will receive any benefits from this study.

PAYMENTS: You will not receive any compensation for your participation.

PARTICIPANT'S RIGHTS: If you have read this form and have decided to participate in this project, please understand your participation is voluntary and you have the right to withdraw your consent or discontinue participation at any time without penalty or loss of benefits to which you are otherwise entitled. The alternative is not to participate. You have the right to refuse to answer particular questions. The results of this research study may be presented at scientific or professional meetings or published in scientific journals. Your individual privacy will be maintained in all published and written data resulting from the study.

CONTACT INFORMATION:

Questions: If you have any questions, concerns or complaints about this research, its procedures, risks and benefits, contact the Protocol Director, Ronald W. Davis.

Phone: +1 650-721-5651; Email: [ron.davis@stanford.edu](mailto:ron.davis@stanford.edu) Alternate contact: Amit Saha

Phone: +1 650-721-5518; Email: [amit.saha@stanford.edu](mailto:amit.saha@stanford.edu)

Independent Contact: If you are not satisfied with how this study is being conducted, or if you have any concerns, complaints, or general questions about the research or your rights as a participant, please contact the Stanford Institutional Review Board (IRB) to speak to someone independent of the research team at (650) 723-5244 or toll free at 1-866-680-2906. You can also write to the Stanford IRB, Stanford University, 1705 El Camino Real, Palo Alto, CA 94306.

Please print a copy of this page for your records.

If you agree to participate in this research, please complete the attached survey.

Please refrain from adding any personal/identifiable information in the survey text boxes.

**Introduction**

Country of Residence United States of America

India China Canada

American Samoa Andorra

Angola Anguilla Antarctica

Antigua and Barbuda Argentina

Armenia Aruba

Ashmore and Cartier Islands Australia

Austria Azerbaijan Bahamas, The Bahrain Bangladesh Barbados Bassas da India Belarus Belgium

Belize Benin Bermuda Bhutan Bolivia

Bosnia and Herzegovina Botswana

Bouvet Island Brazil

British Indian Ocean Territory British Virgin Islands

Brunei Bulgaria Burkina Faso Burma Burundi Cambodia Cameroon Algeria

Cape Verde Cayman Islands

Central African Republic Chad

Chile Albania

Christmas Island Clipperton Island Cocos (Keeling) Islands Colombia

Comoros

Congo, Democratic Republic of the Congo, Republic of the

Cook Islands Coral Sea Islands Costa Rica

Cote d'Ivoire Croatia Cuba Cyprus


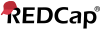
Czech Republic Denmark Dhekelia Djibouti Dominica


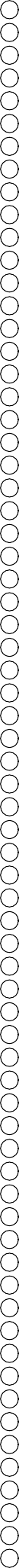
04/27/2021 11:26am

Dominican Repu[pbrloicjectredcap.org](https://projectredcap.org/)


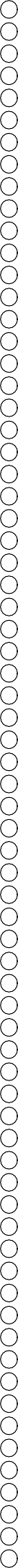
Ecuador Egypt

El Salvador Equatorial Guinea Eritrea

Estonia Ethiopia Europa Island

Falkland Islands (Islas Malvinas) Faroe Islands

Fiji Finland France

French Guiana French Polynesia

French Southern and Antarctic Lands Gabon

Gambia, The Gaza Strip Georgia Germany Ghana Gibraltar Glorioso Islands Greece Greenland Grenada Guadeloupe Guam Guatemala Guernsey Guinea

Guinea-Bissau Guyana

Haiti

Heard Island and McDonald Islands Holy See (Vatican City)

Honduras Hong Kong Hungary Iceland Akrotiri Indonesia Iran

Iraq Ireland Isle of Man Israel

Italy Jamaica Jan Mayen Japan Jersey Jordan

Juan de Nova Island Kazakhstan

Kenya Kiribati Korea, North Korea, South Kuwait Kyrgyzstan Laos

Latvia Lebanon Lesotho Liberia Libya

Liechtenstein Lithuania Luxembourg


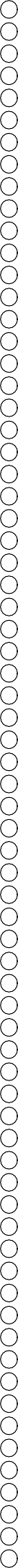
Macau Macedonia Madagascar Malawi Malaysia Maldives Mali

Malta

Marshall Islands Martinique Mauritania Mauritius Mayotte

Mexico

Micronesia, Federated States of Moldova

Monaco Mongolia Montserrat Morocco Mozambique Namibia Nauru

Navassa Island Nepal Netherlands

Netherlands Antilles New Caledonia

New Zealand Nicaragua Niger Nigeria

Niue

Norfolk Island

Northern Mariana Islands Norway

Oman Pakistan Palau Panama

Papua New Guinea Paracel Islands Paraguay

Peru Philippines Pitcairn Islands Poland Portugal Puerto Rico Qatar

Reunion Romania Russia Rwanda Saint Helena

Saint Kitts and Nevis Saint Lucia

Saint Pierre and Miquelon

Saint Vincent and the Grenadines Samoa

San Marino

Sao Tome and Principe Saudi Arabia

Senegal

Serbia and Montenegro Seychelles

Sierra Leone Singapore Slovakia Slovenia Solomon Islands


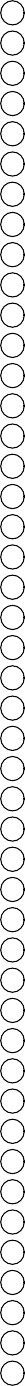
Somalia South Africa

South Georgia and the South Sandwich Islands Spain

Spratly Islands Sri Lanka

218. Sudan Suriname Svalbard Swaziland Sweden Switzerland Syria Taiwan Tajikistan Tanzania Thailand Timor-Leste Togo Tokelau Tonga

Trinidad and Tobago Tromelin Island Tunisia

236. Turkey Turkmenistan

Turks and Caicos Islands Tuvalu

Uganda Ukraine

United Arab Emirates United Kingdom Afghanistan

Uruguay Uzbekistan Vanuatu Venezuela Vietnam Virgin Islands Wake Island

Wallis and Futuna West Bank Western Sahara Yemen

Zambia Zimbabwe


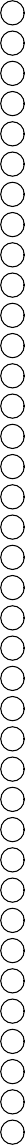
State or Union Territory of Residence in India Andaman and Nicobar Islands Andhra Pradesh

Arunachal Pradesh Assam

Bihar Chandigarh Chhattisgarh

Dadra and Nagar Haveli Daman and Diu

Goa Gujarat Haryana

Himachal Pradesh Jammu and Kashmir Jammu and Kashmir Jharkhand Karnataka

Kerala Ladakh Lakshadweep

Madhya Pradesh Maharashtra Manipur Meghalaya Mizoram Nagaland

Odisha (former Orissa)

Puducherry (former Pondicherry or Pondichéry) Punjab

Rajasthan Sikkim Tamil Nadu Telangana Tripura

Uttar Pradesh Uttarakhand West Bengal Delhi


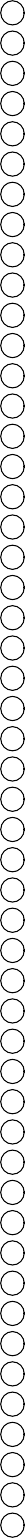
State of Residence in the US Alabama

Alaska Arizona Arkansas California Colorado Connecticut Delaware Florida Georgia Hawaii Idaho Illinois Indiana Iowa Kansas Kentucky Louisiana Maine Maryland

Massachusetts Michigan Minnesota Mississippi Missouri Montana Nebraska Nevada

New Hampshire New Jersey New Mexico New York

North Carolina North Dakota Ohio Oklahoma Oregon Pennsylvania Rhode Island South Carolina South Dakota Tennessee Texas

Utah Vermont Virginia Washington West Virginia Wisconsin Wyoming

District of Columbia / Washington DC

Residence of Province in China Anhui Province Beijing Municipality


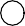

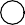

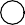

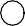

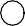

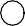

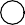

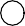

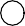

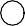

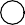

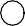

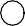

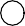

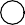

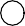

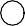

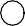

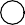

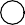

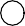

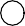

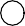

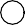

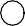

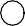

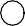

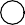

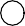

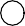

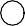

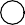

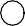

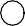


Chongqing Municipality Fujian Province[e] Gansu Province Guangdong Province[g]

Guangxi Zhuang Autonomous Region Guizhou Province

Hainan Province[h] Hebei Province Heilongjiang Province Henan Province

Hong Kong Special Administrative Region Hubei Province

Hunan Province

Inner Mongolia Autonomous Region Jiangsu Province

Jiangxi Province Jilin Province Liaoning Province

Macau Special Administrative Region Ningxia Hui Autonomous Region Qinghai Province

Shaanxi Province Shandong Province Shanghai Municipality Shanxi Province Sichuan Province Taiwan Province[l] Tianjin Municipality

Tibet Autonomous Region

Xinjiang Uyghur Autonomous Region Yunnan Province

Zhejiang Province

State of Residence

Age bracket (in years) 18 - 25

26 - 35


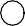

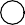

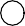

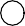

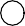

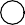

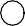


36 - 45

46 - 55

56 - 65

66 - 75

>75

Race and Ethnicity American Indian / Alaska Native Asian

Native Hawaiian or Other Pacific Islander Black or African American


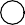

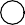

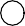

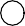

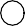

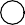

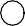

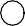


White

Hispanic or Latino More Than One Race Prefer not to answer


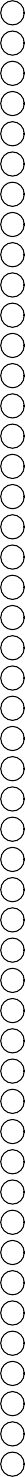
Specify the region in Asia Afghanistan

Armenia Azerbaijan Bahrain Bangladesh Bhutan Brunei Cambodia China Cyprus Georgia India Indonesia Iran

Iraq Israel Japan Jordan

Kazakhstan Kuwait Kyrgyzstan Laos Lebanon Malaysia Maldives Mongolia Myanmar Nepal

North Korea Oman Pakistan Philippines Qatar

Saudi Arabia Singapore South Korea Sri Lanka

State of Palestine Syria

Tajikistan Thailand Timor-Leste Turkey Turkmenistan

United Arab Emirates Uzbekistan

Vietnam Yemen Other

Gender Female

Male Transgender Other


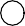

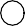

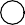

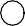

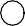


Prefer not to answer

Other

Choose current status from list below to the best of Single


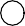

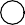

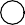

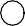

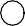

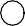

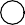


your knowledge Domestic relationship / Living with partner Married

Separated Divorced Widowed

Prefer not to answer

People you live with (check all that apply)

Spouse or partner Children

Parents Siblings

By yourself Other relatives Friends

Housemates / Roommates Prefer not to answer

Number of people currently living in your household 1-2


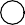

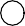

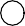

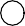


(including yourself) 3-5

5-8

>8

Highest degree of education Less than high school High school or equivalent Associate or equivalent Bachelors

Masters


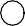

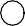

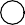

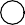

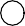

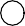

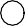


Doctoral / Professional Prefer not to answer

To your best knowledge, please choose income group to Low

the nearest category Lower middle


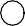

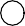

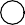

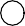

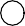

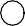


Middle

Upper middle High

Prefer not to answer

Employment status (check all that apply)

Employed Full-time

Self-employed / Freelance Employed Part-time Unemployed

Homemaker Student Retired

Prefer not to answer

Unemployed

(check all that apply)

Looking for work Not looking for work

Lost work due to COVID-19

Choose the category that best fits your employment Healthcare Finance


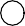

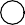

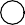

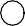

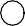

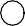

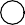

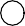

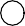

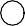

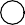

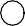

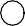

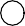


Science & Technology Education

Research

Biotechnology & Pharmaceutical Transportation

Local business News & Media

Art & Entertainment Military/Forces

First Responders Civil Services Other

Please specify 'Other'

Are you an essential worker during COVID-19 pandemic? Yes

No

Prefer not to answer

Tell us about your work schedule as an essential I have to go to work every day

worker I have to go 2-3 times a week

I have to go as necessary

I don't have to go and can work from home

Tell us about your schedule as a non-essential worker I work from home

I cannot work from home N/A

Have you been tested for COVID-19? Yes No

Prefer not to answer

Result of the test Positive

Negative

Prefer not to answer

Type of sample submitted for the test Blood Nasal Swab Both 1 & 2

Prefer not to answer

Current quarantine status Hospital / Clinic

Home

Some other location

Currently, has anyone in your immediate friends/family been tested positive for COVID-19?

Yes. My household

Yes. Immediate family not in my household Yes. Friend(s)

Yes. My neighbor(s) None I know

Prefer not to answer

To the best of your knowledge, what percentage of your Rarely (0-20%) daily total interaction with anyone involves talking A little bit (21-40%)

about COVID-19? About half the time (41-60%)

A lot (>60%)

Prefer not to answer

# Social Interactions

Part 2 out of 5

During COVID-19 pandemic, do you see any change in Yes your social interaction with non-household friends & No

family? Not sure

Prefer not to answer

What social changes have you made to cope with the pandemic?

(check all that apply)

Checking up on family / friends

Revival of long lost connections with family / friends

Other

Prefer not to answer

Other

How do you feel about spending more time in your I live by myself

household during this pandemic? Great Neutral

Overwhelming It varies

Prefer not to answer

After end of this pandemic, do you intend to maintain Yes, definitely similar level of social interactions with friends & Maybe, I will try to

family? No, it's overwhelming

Prefer not to answer

**During social distancing, how are you communicating with friends/family who are not in your**

**household?**

Video call Audio call

Messaging (imsg / Whats app / Messenger )

Email

Social Media (Facebook / Twitter

/ Instagram / TikTok)

Video Conferencing Other

Rarely (0-20%) A little bit

(21-40%)

About half the

time (41-60%)

A lot (>60%) Prefer not to

answer

Other

During this pandemic, did you feel the need to Yes

transition from audio calls to video calls to No

communicate with friends & family? Prefer not to answer

# Hobbies and Health

Part 3 out of 5

Have you discovered any new hobbies? (check all that apply)

Yes No

Still exploring Revisiting an old hobby Prefer not to answer

**How are you utilizing your personal time?**

Household work / Home improvements

Cooking / becoming Masterchef

:G)ardening Reading / Writing Indoor & Games Performing Arts Online learning

Watching Movies / DArotcsu&mCernatfatrsies

Photography & Videography Fitness & Health

Spending time with kids Other

Rarely (0-20%) A little bit

(21-40%)

About half the time (41-60%)

A lot (>60%) Prefer not to

answer

Other

**What is the effect of stay-at-home order on your overall health?**

Physical Health Mental Health

Socio-emotional Health

Great Neutral Could have been better

It varies Prefer not to answer

Before COVID-19, on an average how much time did you More than 4 times a week spend on physical activities? Between 3-4 times a week Between 1-2 times a week

(run / walk / exercises / gardening / yoga /golf etc) Between 2-4 times a month

Rarely (less than once a month) I cannot do any physical activity Prefer not to answer

| Since the start of COVID-19, on an average how much |  | More than 4 times a week |
| --- | --- | --- |
| time did you spend on physical activities? |  | Between 3-4 times a week |
|  |  | Between 1-2 times a week |
| (run / walk / exercises / gardening / yoga /golf etc) |  | Between 2-4 times a month |
|  |  | Rarely (less than once a month) |
|  |  | I cannot do any physical activity |
|  |  | Prefer not to answer |
| What is the effect of COVID-19 lockdown on your mental |  | I am extremely affected |
| health? |  | I am moderately affected |
|  |  | It hasn't affected me at all |
|  |  | It has not affected but I feel anxious at times |
|  |  | It's overwhelming |
|  |  | It fluctuates depending on my mood |
|  |  | Prefer not to answer |
| How are you coping with your mental stress? |  | I talk to friends/family |
| (check all that apply) |  | I go to online therapy sessions |
|  |  | I do meditation/yoga |
|  |  | I pursue my hobbies |
|  |  | I perform religious practices |
|  |  | Other |
|  |  | Prefer not to answer |
| Please specify 'Other' |  |  |
|  |  |  |
| Health Professionals recommend frequent hand washing |  | Yes. I am washing hands more than before |
| and improve hygiene / habits. Do these have any effect |  | Yes. I shower every time I get home from outside |
| on you? |  | Yes. I keep wiping down all the common areas |
| (check all that apply) |  | No. I have just been social distancing |
|  |  | Prefer not to answer |
| **Have you ever been diagnosed with the following?** |  |  |
| Yes | No | Not aware of the term Prefer not to answer |

Diabetes Cardiovascular Disorders Obesity

Respiratory Infections (Example: flu, tuberculosis, pneumonia)

Respiratory Disorders (Example: asthma, bronchitis, cystic

fibrosis)

Gastrointestinal Disorders

Chronic Kidney Disease Autoimmune Disease

Myalgic Encephalomyelitis / Chronic Fatigue Syndrome

(ME/CFS)

**Are you aware that a viral infection can cause:**

Yes No Not aware of the term Prefer not to answer

Skin Warts Type 1 Diabetes Liver Cirrhosis Seasonal flu / Influenza

Myalgic Encephalomyelitis / Chronic Fatigue Syndrome

(ME/CFS)

**Please rate the following to the best of your knowledge:**

**1 = least affected 5 = most affected**

1 2 3 4 5

Overall daily routine Physical Health Mental Health Time spent with family Time spent working Time spent on hobbies

# Professional Life

Part 4 out of 5

Does the nature of your work allow you to work from Yes. I can work from home entirely without any home during this lockdown period? effect

No. I cannot work from home at all

Partially affected (Part of my work requires me to go onsite)

I do not work

Prefer not to answer

How well are you able to balance work life and Excellent

personal/family life? Good

Neutral

Could be improved Prefer not to answer

Did you lose job during this pandemic era? Yes No

Prefer not to answer

Did COVID-19 affect your income even though your job Yes is secure? No

Partially

Prefer not to answer

Are you actively looking for a new job? Yes No

Prefer not to answer

How well are you utilizing your time now to work on your professional skills?

(1 being the worst and 5 being the best)

1 2 3 4

5

Are you looking for a career transition at this time? Yes No

I don't work

Prefer not to answer

Professionally, are you pursuing any of the following options to grow in your career?

Networking with my peers

Online training on professional development courses Trying to switch my area of expertise

Happy with my current position N/A

Other

Other

If you are a full-time/part-time student, do you see Yes

any impact on your studies? No

Partially N/A

How has it affected your studies? (Choose all that apply)

Lost focus on studies

Online learning isn't effective Internet isn't accessible to me

More guidance from teachers needed Cannot concentrate on studies at home Prefer not to answer

# Social contributions

Part 5 out of 5

In your experience, do you think social distancing 1

affected volunteering efforts to help the community? 2

3

1 = least affected 4

5 = most affected. 5

Did you contribute to help your local community in Yes

these unprecedented times? No

Prefer not to answer

Do you plan to contribute in the future? Yes No

Maybe Yes Maybe No

How did you help your local or global community? (check all that apply)

Donated my time/efforts Volunteered at NGOs/NPOs Provided financial assistance

Provided blood/food/groceries/in-kind donations Other

Other

Where have your contributions helped? (check all that apply)

In health care sector

For elderly people and families who have losses due to the pandemic

To fundraise for low income families For animal welfare/pet relief funds Other

Other
